# Enhanced computed tomography radiomics predicts solute carrier family 7, member 11 in head and neck squamous cell carcinoma

**DOI:** 10.3389/fgene.2024.1418578

**Published:** 2024-09-16

**Authors:** Jilian Lv, Xiangze Meng, Yuanyuan Li, Rui Zhang, Yuan Zhao, Xi Yang, Fang Wang, Xinbin Wang

**Affiliations:** ^1^ Department of Oral and Maxillofacial Surgery, Zhengzhou Central Hospital Affiliated to Zhengzhou University, Zhengzhou, China; ^2^ Department of Oromaxillofacial Head and Neck Oncology, Shanghai Ninth People’s Hospital, Shanghai Jiao Tong University School of Medicine, Shanghai, China; ^3^ Department of Oral and Maxillofacial Surgery, Lishui Central Hospital, Lishui Hospital of Zhejiang University, Lishui, China; ^4^ Department of Oral Surgery, Shanghai Ninth People’s Hospital, Shanghai Jiao Tong University School of Medicine, Shanghai, China

**Keywords:** head and neck squamous cell carcinoma, SLC7A11, enhanced CT, radiomics, clinical prognosis

## Abstract

**Introduction:**

Traditional prognostic indicators for head and neck squamous cell carcinoma (HNSCC), such as clinicopathological features, human papillomavirus status, and imaging examinations, often lack precision in guiding medical therapy. Therefore, discovering novel tumor biomarkers that can accurately assess prognosis and aid in personalized medical treatment for HNSCC is critical. Solute carrier family 7, member 11 (SLC7A11), is implicated in ferroptosis, and various malignant tumor therapies regulate its expression. However, the mechanisms regulating SLC7A11 expression, the transporter activity, and its specific role in controlling ferroptosis in cancer cells remain unknown. Thus, in this study, we aimed to develop an improved computed tomography (CT) radiomics model that could predict SLC7A11 expression in patients with HNSCC.

**Methods:**

We used patient genomic data and corresponding augmented CT images for prognostic analysis and building models. Further, we investigated the potential molecular mechanisms underlying SLC7A11 expression in the immune microenvironment. Our radiomics model successfully predicted *SLC7A11* mRNA expression in HNSCC tissues and elucidated its association with relevant genes and prognostic outcomes.

**Results:**

SLC7A11 expression level was high within tumor tissues and was connected to the infiltration of eosinophil, CD8^+^ T-cell, and macrophages, which was associated with poor overall survival. Our models demonstrated robust predictive power. The distribution of radiomics scores (RAD scores) within the training and validation sets was markedly different between the high- and low-expression groups of SLC7A11.

**Conclusion:**

SLC7A11 is likely an important factor in the prognosis of HNSCC. SLC7A11 expression can be predicted effectively and reliably by radiomics models based on enhanced CT.

## 1 Introduction

Over 90% of all head and neck malignant tumors are caused by head and neck squamous cell carcinoma (HNSCC), which has an annual incidence of 6,00,000 (Bray et al., 2018). Furthermore, HNSCC is associated with mortality rates ranging from 40% to 50% (Leemans et al., 2018; Zhou et al., 2021), resulting in it being the sixth most common cancer worldwide (Johnson et al., 2020). Metastasis significantly contributes to the morbidity of HNSCC, and its presence strongly influences treatment decisions (Cramer et al., 2019; Chow, 2020). However, the molecular mechanisms underlying HNSCC invasion and metastasis remain unclear. Traditional prognostic indicators, including clinicopathological features, human papillomavirus status, computed tomography (CT), and magnetic resonance imaging (MRI), frequently fail to provide enough information to guide precise medical therapy. Therefore, identifying novel tumor biomarkers is essential for efficiently evaluating HNSCC prognosis and providing personalized medical treatment.

SLC7A11 is a gene that encodes proteins and belongs to the solute carrier family 7, member 11. This gene mediates the transport of cysteine and glutamate independent of sodium ions (Lin et al., 2020). Kaposi sarcoma-associated herpes viruses can also be fused and entered through the action of SLC7A11 (Kaleeba and Berger, 2006). Further, *SLC7A11* is involved in various signaling pathways, including glucose, bile, and metal ion transport, and responds to increased cytoplasmic Ca^2+^ levels (Lin et al., 2020). Furthermore, *SLC7A11* is implicated in ferroptosis, a novel cell death mechanism, and its expression is regulated by malignant tumor therapies, such as immunotherapy and radiation therapy (Koppula et al., 2021). However, the precise regulatory mechanisms governing *SLC7A11* expression and transporter activity and its specific role in controlling ferroptosis in cancer cells remain unknown, and these gaps in knowledge have attracted widespread attention in the scientific community (Feng et al., 2021). Related studies revealed that expression of SLC7A11, regulated by nuclear factor-erythroid 2 related factor 2, decreases the radiosensitivity of esophageal squamous cell carcinoma through the suppression of ferroptosis. Moreover, *SLC7A11* upregulates programmed death ligand 1 (*PD-L1*) and colony-stimulating factor 1 (He et al., 2021), highlighting its clinical significance.

Omics technologies have become widely accepted as a valuable tool for the diagnosis and treatment of HNSCC; however, invasive tissue biopsies often fail to capture tumor heterogeneity. Radiomics, a noninvasive technique, enables comprehensive tumor assessment by segmenting and outlining regions of interest in medical imaging data, extracting numerous feature parameters using automated algorithms, and analyzing clinical phenotypes. Techniques encompassed by radiomics include such as ultrasonography, radiography, CT, MRI, or positron emission tomography. Radiomics holds promise for assessing patient genotypes, treatment efficacy, and clinical outcomes (Peng et al., 2021).

This study aimed to use an enhanced CT radiomics model to noninvasively evaluate and forecast SLC7A11 mRNA levels in HNSCC tissue samples. Furthermore, we aimed to examine the link between the radiomics score (RAD score) and ferroptosis-related gene expression and to identify the potential molecular biology mechanism of *SLC7A11* in the immune microenvironment through bioinformatics analysis.

## 2 Materials and methods

### 2.1 Data processing and bioinformatics analysis

We noninvasively evaluated and predicted *SLC7A11* mRNA levels in HNSCC tissue samples by incorporating and screening primary solid tumor data from the Cancer Genome Atlas regarding HNSCC using transcriptome sequencing data. RNA-sequencing data were processed through a tour process using UCSC Xena software.

### 2.2 Survival analysis

Kaplan–Meier survival curves were generated to estimate changes in survival rate using landmark analysis over time, with 12, 24, 36, 48, and 60 months used as the time nodes post-surgery for HNSCC. The period between the initial diagnosis and the time node was defined as “early,” and that between the time node until the end of the follow-up period was defined as “late.” The Kaplan–Meier curves plotted based on the landmark analysis show the survival time on the *x*-axis and mortality risk on the *y*-axis.

### 2.3 Univariate and multivariate cox regression analyses

Overall survival (OS) was the primary outcome variable, and SLC7A11 expression was the main independent variable. The threshold value was calculated using the “survivor” package. The HNSCC tissue samples were divided into groups with high- and low-expression levels, with the latter being the reference group. SLC7A11 expression on patient prognosis in various subgroups was investigated using univariate Cox regression analysis.

### 2.4 Correlation analysis of immune cell infiltration

Using the CIBERSORTx database, we calculated immune cell infiltration in each HNSCC sample and analyzed the correlation between *SLC7A11* expression and immune cell infiltration.

### 2.5 Enrichment analysis

The Kyoto Encyclopedia of Genes and Genomes (KEGG) pathways and hallmark gene sets were enriched using gene-set variation analysis for each sample.

### 2.6 Image target segmentation and feature extraction

The Cancer Genome Atlas HNSCC data and arterial phase-enhanced CT images in the TCIA data (https://www.cancerimagingarchive) were analyzed. Cases with missing survival data and clinical transcriptome variables that were missing or had a survival time of less than 30 days were excluded from the list. Postoperative images with artifacts in the target area were excluded from the imaging data. The regions of interest in the images were defined as the tumor areas, which were identified using naked-eye observations. Two physicians independently applied three-dimensional slicer software to segment the regions of interest of the images from 30 patients and extracted radiomic features using the “Pyradiomics” package. An interclass correlation coefficient analysis was performed after the target area was delineated. After verifying consistency, one of the physicians completed the segmentation and feature extraction of the remaining samples.

### 2.7 Image feature filtering

In a ratio of 7:3, the target area image data were randomly divided into training and validation sets, respectively, and an intergroup difference analysis of the two sets was performed. Features with a variance of zero and strong correlations (>0.9) were removed, and a recursive feature elimination algorithm was used to obtain the optimal feature subset.

### 2.8 Building a radiomics prediction model

Support vector machines (SVM) is a binary classification model. The basic model is to define a linear classifier with the largest interval in the feature space, and to find a high latitude hyperplane as the decision boundary through support vectors. The SVM algorithm is used to model the screened image genomics features, which are used to predict gene expression.

Logistic regression (LR) transforms linear regression through the Sigmoid function, making the model output values distributed between (0,1) and (0,1). The LR algorithm uses the stats package “glm” to fit the screened image histological features. On this basis, the established LR model was subjected to two-way stepwise regression for further feature selection, and based on the AIC criterion, the feature group with the smallest AIC was selected to construct the final model for predicting the expression of *SLC7A11*. Rad score = Feature* corresponding coefficients (Estimate) + Intercept value (Estimate).

LR and SVM algorithms were employed to construct radiomic models for predicting gene expression. The model with the best predictive performance was chosen and executed. To predict gene expression levels, we devised the RAD score.

### 2.9 Evaluation of the radiomics prediction models

**FIGURE 1:**
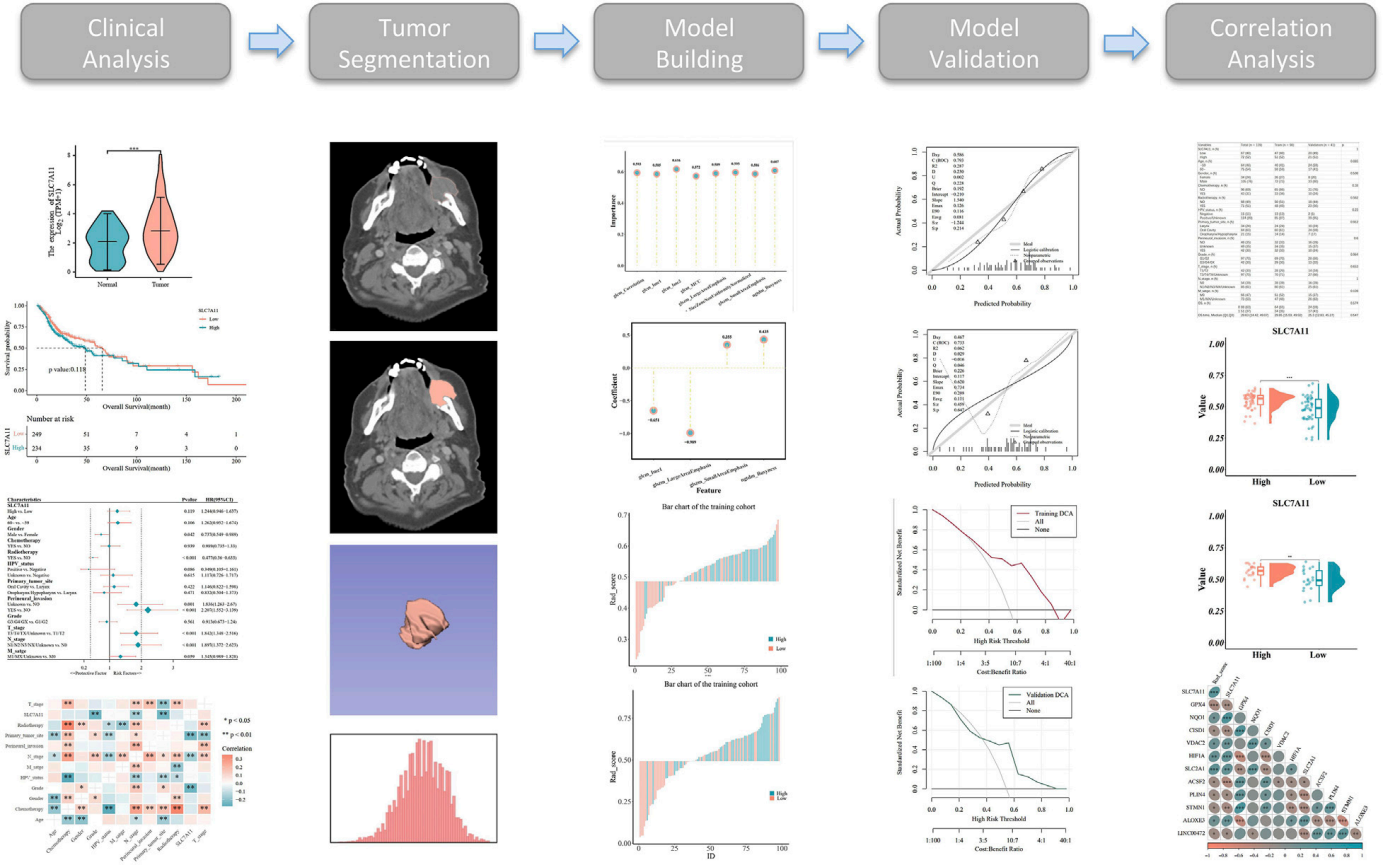
Workflow used in the study to identify a radiomics-related prognostic signature and perform correlation analysis in head and neck squamous cell carcinoma (HNSCC).

The model evaluation indicators included accuracy, specificity, sensitivity, positive predictive values, negative predictive values, receiver operating characteristic (ROC) curves, precision–recall curves, Hosmer–Lemeshow goodness-of-fit test, Brier scores, decision curve analysis, and the DeLong test (Figure 1).

### 2.10 Statistical analysis

We used SPSS version 23.0 to perform all statistical and data analyses. Intergroup difference analysis was performed using " ggplot2”. “Survival” and “Survivor” packages were utilized for survival analysis. Subgroup and interaction analyses were performed using “cmprsk,” “survival,” and “forest plots” packages. Correlation analyses involved performing Pearson’s correlations. Enriching gene-set variations was achieved using Limma and gene-set analysis. Consistency was evaluated using the “irr” package. “Caret” and “CBCgrps” packages were used for dataset partitioning. “Caret statistics” and “MASS” were used for feature filtering and model construction, respectively. “PROC,” “measures,” “resource selection,” “rms,” and “rmda” packages were used for model evaluation. Statistical significance was defined by a *p*-value > 0.05.

## 3 Results

### 3.1 Patient characteristics

The survival study involved 483 HNSCC patient samples from the Cancer Genome Atlas database. The samples were separated into two groups: low-expression (n = 249) and high-expression (n = 234) groups. The truncation value was 1.620258. The clinical data of the patients analyzed in this study can be found in Table 1. Significant differences in the human papillomavirus status, perivascular infiltrate distribution, radiotherapy, and primary tumor site location were observed between the high- and low-expression groups of SLC7A11.

**TABLE 1:** Patient characteristics.

Variables	Total (n = 483)	Low (n = 249)	High (n = 234)	*p*
Age	n (%)			0.217
≤59 years	211 (44)	116 (47)	95 (41)	
≥60 years	272 (56)	133 (53)	139 (59)	
Sex	n (%)			0.916
Female	128 (27)	67 (27)	61 (26)	
Male	355 (73)	182 (73)	173 (74)	
Chemotherapy	n (%)			0.385
No	322 (67)	161 (65)	161 (69)	
Yes	161 (33)	88 (35)	73 (31)	
Radiotherapy	n (%)			0.451
No	234 (48)	116 (47)	118 (50)	
Yes	249 (52)	133 (53)	116 (50)	
HPV status	n (%)			<0.001
Negative	68 (14)	27 (11)	41 (18)	
Positive	30 (6)	25 (10)	5 (2)	
Unknown	385 (80)	197 (79)	188 (80)	
Primary tumor site	n (%)			<0.001
Larynx	109 (23)	45 (18)	64 (27)	
Oral cavity	297 (61)	144 (58)	153 (65)	
Oropharynx/hypopharynx	77 (16)	60 (24)	17 (7)	
Perineural invasion	n (%)			0.44
No	181 (37)	89 (36)	92 (39)	
Unknown	141 (29)	79 (32)	62 (26)	
Yes	161 (33)	81 (33)	80 (34)	
Grade	n (%)			<0.001
G1/G2	348 (72)	160 (64)	188 (80)	
G3/G4/GX	135 (28)	89 (36)	46 (20)	
T stage	n (%)			0.231
T1/T2	173 (36)	96 (39)	77 (33)	
T3/T4/TX/unknown	310 (64)	153 (61)	157 (67)	
N stage	n (%)			0.007
N0	164 (34)	70 (28)	94 (40)	
N1/N2/N3/NX/unknown	319 (66)	179 (72)	140 (60)	
M stage	n (%)			0.82
M0	174 (36)	88 (35)	86 (37)	
M1/MX/unknown	309 (64)	161 (65)	148 (63)	

Abbreviation: HPV, human papillomavirus.

### 3.2 Relationship between gene expression levels and clinical features

**FIGURE 2:**
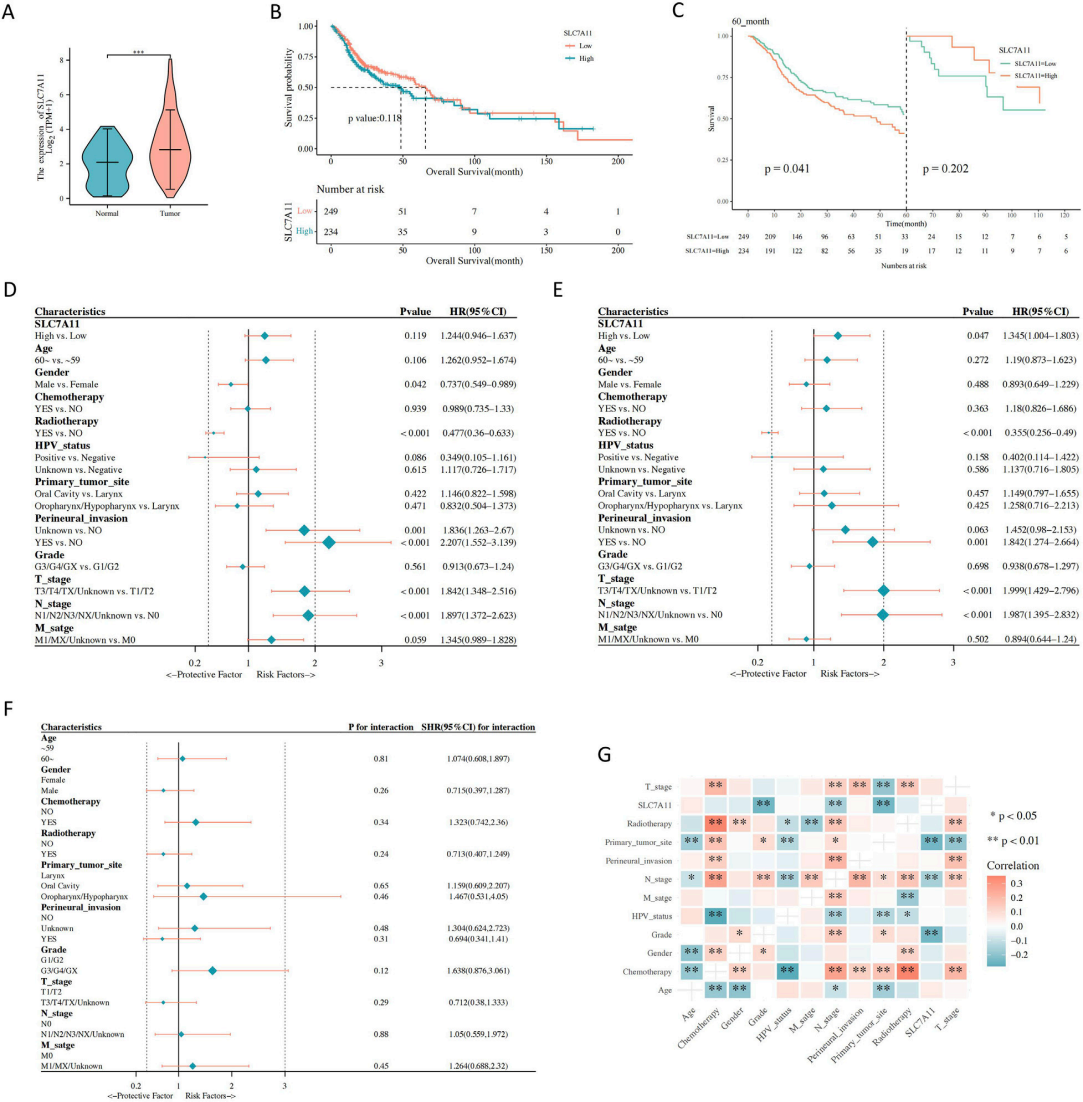
The association between gene expression and clinical characteristics. **(A)** Solute carrier family 7, member 11 (*SLC7A11*) expression in normal and tumor tissues. **(B)** Correlations between *SLC7A11* expression and the overall survival (OS) of patients with head and neck squamous cell carcinoma (HNSCC). **(C)** Landmark analysis. **(D)** Univariate Cox regression analyses. **(E)** Multivariate Cox regression analyses. **(F)** Subgroup analysis and interaction test. **(G)** Correlation analysis between *SLC7A11* expression and clinical parameters of HNSCC.

*SLC7A11* expression was high in tumor tissues (Figure 2A). The low and high *SLC7A11* expression groups had median survival durations of 48.63 and 65.73 months, respectively. The Kaplan–Meier curve analysis revealed that elevated *SLC7A11* expression strongly correlated with improved OS (Figure 2B). Additionally, sex, human papillomavirus status, T stage, N stage, perivascular infiltrates, and radiation therapy were significantly associated with higher OS (Supplementary Figure S1A–F). The landmark analysis was performed 60 months after the HNSCC diagnosis. Higher SLC7A11 expression was related to poorer survival rates during the early stages. However, during the late stage, OS did not show a significant difference between the high and low SLC7A11 expression groups (Figure 2C). High SLC7A11 expression was linked to poor OS in the univariate analysis. Additionally, sex and radiation therapy were protective factors for OS (Figure 2D). After adjusting for other factors, the multivariate analysis showed that the expression of SLC7A11 remained a significant prognostic factor. However, radiation therapy continued to be a protective factor for OS (Figure 2E). Poor OS was found to be a risk factor due to increased SLC7A11 expression during the G3/G4/GX subgroup analysis (Figure 2F). *SLC7A11* expression was significantly associated with the tumor grade, initial tumor site, and N stage (Figure 2G).

### 3.3 *SLC7A11* expression affects immune cell infiltration in HNSCC

**FIGURE 3:**
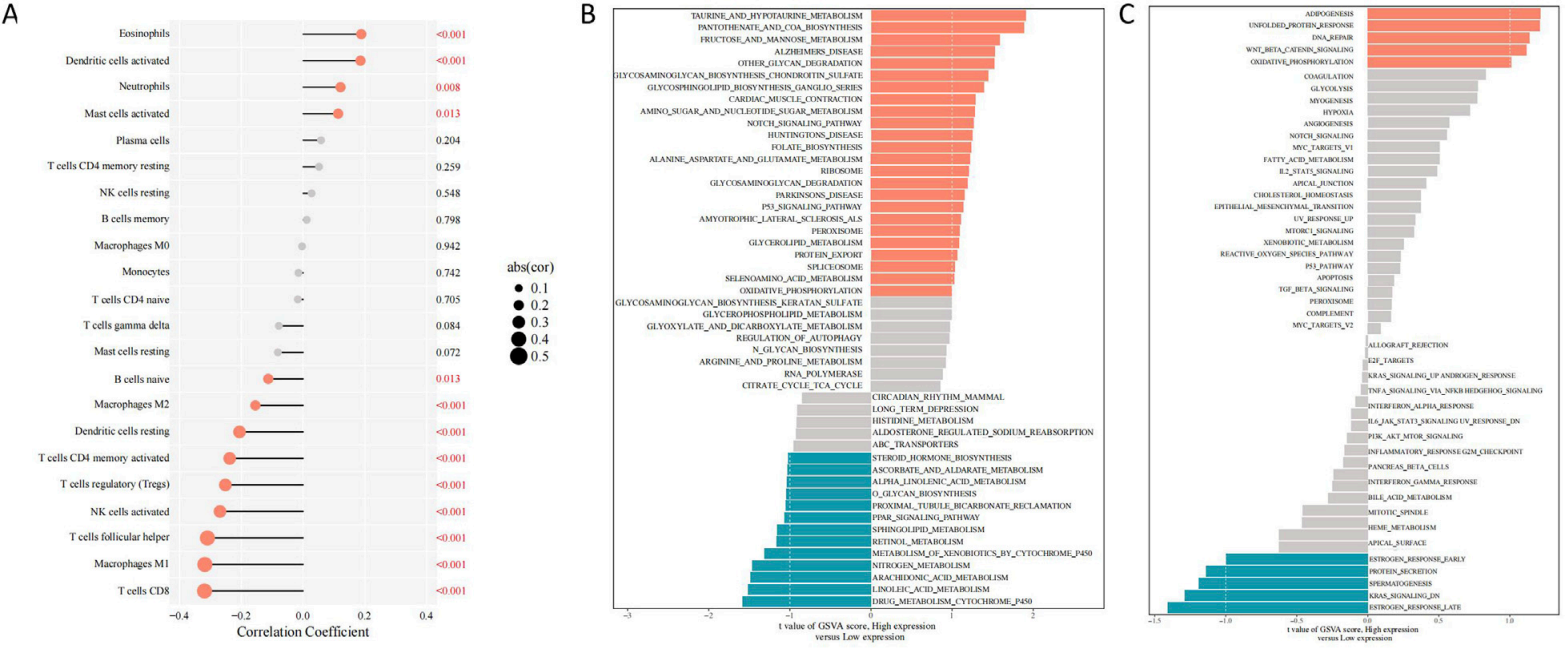
Differentially expressed genes and immune cell infiltration linked to SLC7A11 expression in HNSCC. **(A)** Correlations between *SLC7A11* expression and immune cell infiltration in HNSCC. **(B)** KEGG enrichment analysis. **(C)** Hallmark enrichment analysis. HNSCC, head and neck squamous cell carcinoma; *SLC7A11*, solute carrier family 7, member 11; KEGG, Kyoto Encyclopedia of Genes and Genomes.

According to our findings, the high SLC7A11 expression group showed a increase in the level of 4 types of immune cells infiltration (*p* < 0.05). The immune cell infiltration of eosinophils and dendritic cells activated was significantly positive associated with the high SLC7A11 expression (*p* < 0.001). Further, We found 9 types of immune cells was significantly negatively associated with SLC7A11 expression (*p* < 0.05). The immune cell infiltration of T cells follicular helper, macrophages M1 and T cells CD8 was significantly negatively associated with the low SLC7A11 expression (*p* < 0.001) (Figure 3A).

### 3.4 Differentially expressed genes (DEGs) in the groups with high and low *SLC7A11* expression

The p53 signaling network was significantly enriched with DEGs from the high SLC7A11 expression group, as demonstrated by the KEGG enrichment analysis. In contrast, the peroxisome proliferator-activated receptor signaling pathway was more prevalent among those in the low SLC7A11 expression group (Figure 3B). The hallmark enrichment analysis demonstrated that DEGs in the Wnt/β-catenin and the high SLC7A11 expression group had higher levels of proteins involved in DNA repair signaling pathways, while the low SLC7A11 expression group had lower levels of proteins involved in KRAS signaling pathways (Figure 3C).

### 3.5 Patient clinicopathological features and radiomic analysis

**FIGURE 4:**
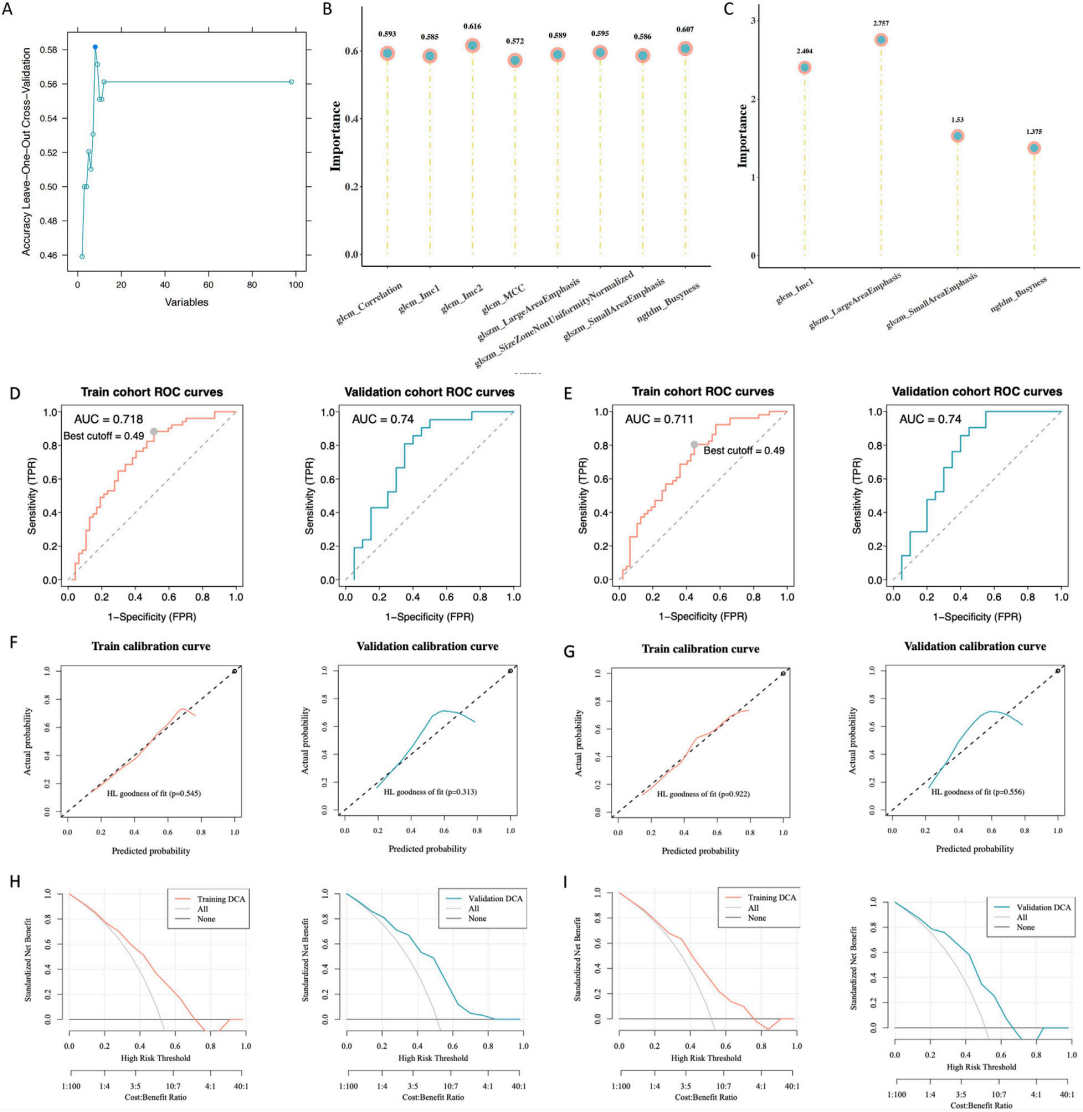
Building and assessing radiomics models. **(A)** Radiomic features with variations in statistics. **(B)** Significance of the chosen characteristics in the support vector machines (SVM) model. **(C)** Significance of the selected features in the logistic regression (LR) model. **(D)** Receiver operating characteristic (ROC) curve analysis of the SVM model. **(E)** ROC curve analysis of the LR model. **(F)** Calibration curve analysis of the SVM model. **(G)** Calibration curve analysis of the LR model. **(H)** Hosmer–Lemeshow goodness-of-fit testing of the SVM model. **(I)** Hosmer–Lemeshow goodness-of-fit testing of the LR model.

The entire dataset was split into a training set with 98 patients and a verification set with 41 patients (Table 2). When analyzing intergroup differences, the baseline conditions of the patients in both sets remained the same and demonstrated that the two groups were comparable (*p* > 0.05). In addition, eight radiomic features were identified (Figure 4A).

**TABLE 2:** Data set division.

Variables	Total (n = 139)	Train (n = 98)	Validation (n = 41)	*p*
SLC7A11	n (%)			1
Low	67 (48)	47 (48)	20 (49)	
High	72 (52)	51 (52)	21 (51)	
Age	n (%)			0.085
≤59 years	64 (46)	40 (41)	24 (59)	
≥60 years	75 (54)	58 (59)	17 (41)	
Sex	n (%)			0.508
Female	34 (24)	26 (27)	8 (20)	
Male	105 (76)	72 (73)	33 (80)	
Chemotherapy	n (%)			0.38
No	96 (69)	65 (66)	31 (76)	
Yes	43 (31)	33 (34)	10 (24)	
Radiotherapy	n (%)			0.562
No	68 (49)	50 (51)	18 (44)	
Yes	71 (51)	48 (49)	23 (56)	
HPV status	n (%)			0.23
Negative	15 (11)	13 (13)	2 (5)	
Positive/unknown	124 (89)	85 (87)	39 (95)	
Primary tumor site	n (%)			0.912
Larynx	34 (24)	24 (24)	10 (24)	
Oral cavity	84 (60)	60 (61)	24 (59)	
Oropharynx/hypopharynx	21 (15)	14 (14)	7 (17)	
Perineural invasion	n (%)			0.6
No	48 (35)	32 (33)	16 (39)	
Unknown	49 (35)	34 (35)	15 (37)	
Yes	42 (30)	32 (33)	10 (24)	
Grade	n (%)			0.964
G1/G2	97 (70)	69 (70)	28 (68)	
G3/G4/GX	42 (30)	29 (30)	13 (32)	
T stage	n (%)			0.653
T1/T2	42 (30)	28 (29)	14 (34)	
T3/T4/TX/unknown	97 (70)	70 (71)	27 (66)	
N stage	n (%)			1
N0	54 (39)	38 (39)	16 (39)	
N1/N2/N3/NX/unknown	85 (61)	60 (61)	25 (61)	
M stage	n (%)			0.139
M0	66 (47)	51 (52)	15 (37)	
M1/MX/unknown	73 (53)	47 (48)	26 (63)	
OS	n (%)			0.574
0	88 (63)	64 (65)	24 (59)	
1	51 (37)	34 (35)	17 (41)	

Abbreviations: HPV, human papillomavirus; OS, overall survival.

### 3.6 Radiomic model construction and evaluation

According to the law of minimum AIC, we further filtered from 8 radiomics features and obtained 4 features to construct the LR model. LR rad_score = (original_glcm_Imc1) * 0.651 + (original_ngtdm_Busyness) * 0.435 + (original_glszm_SmallAreaEmphasis) * 0.355 + (original_glszm_LargeAreaEmphasis) * 0.989 + 0.049.

We developed SVM and LR radiomics models and assessed the significance of selected features (Figures 4B, C). The ROC analysis showed that the radiomic models had good predictive power. For the training and validation sets, the SVM area under the curve (AUC) values were 0.718 and 0.74, respectively (Figure 4D); the corresponding LR AUC values were 0.711 and 0.74, respectively (Figure 4E). The calibration curve analysis (Figures 4F, G) and Hosmer–Lemeshow goodness-of-fit test (Figures 4H, I) indicated that the models accurately predicted gene expression levels, aligning closely with the true values. The decision curve analysis showed that these models have clinical utility. The validation and training sets (SVM, *p* = 0.818; LR, *p* = 0.766) indicated that the models fit well. Furthermore, the interclass correlation coefficients of all selected radiomic features were >0.8, suggesting that these radiomic features had good agreement (Table 3).

**TABLE 3:** Radiomics features.

Item	Importance
original_glcm_MCC	0.977443127
original_glcm_Imc2	0.975515534
original_glcm_Imc1	0.995165417
original_glszm_SizeZoneNonUniformityNormalized	0.972323605
original_ngtdm_Busyness	0.846606774
original_glcm_Correlation	0.969246820
original_glszm_SmallAreaEmphasis	0.896954731
original_glszm_LargeAreaEmphasis	0.947796442

### 3.7 Correlation analysis

**FIGURE 5:**
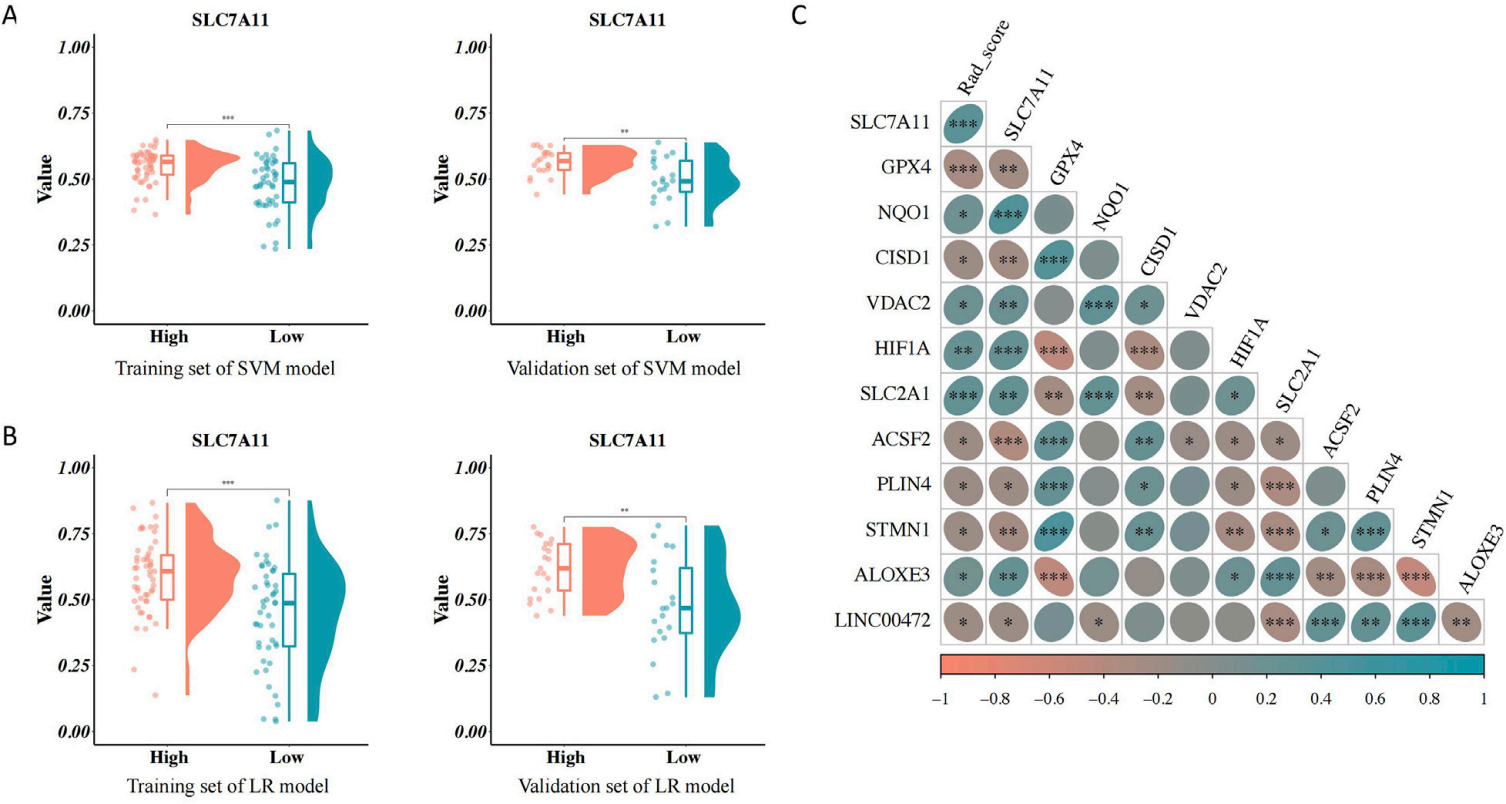
Association between the RAD score and clinical characteristics. **(A)** Association between the RAD score of the support vector machines (SVM) model and solute carrier family 7, member 11 (*SLC7A11*) expression. **(B)** Association between the RAD score of the logistic regression model and *SLC7A11* expression. **(C)** Correlation analysis of the RAD score, *SLC7A11* expression, and ferroptosis-related genes.

The distribution of the RAD scores in the two sets of the SVM and LR models varied significantly between the different *SLC7A11* expression groups (*p* < 0.05). The RAD score was higher in the group with high *SLC7A11* expression (Figures 5A, B). Furthermore, *SLC7A11* expression and the RAD score were positively related to the gene expression levels of *NQO1*, *VDAC2*, *HIF1A*, *SLC2A1*, and *ALOXE3* (Figure 5C).

## 4 Discussion

One of the major purposes of this study was to use an enhanced CT radiomics model to forecast the expression of *SLC7A11* in HNSCC and evaluate its clinical prognostic significance. Our investigation showed a correlation between tumor grading and SLC7A11 expression. Imaging feature discrepancies between the different *SLC7A11* expression groups were verified, along with the validation of the construction of SVM and LR prediction models. Further, *SLC7A11* expression and the RAD score positively correlated with ferroptosis-related genes (correlation coefficients: 0.270 and 0.307, respectively; *p* < 0.01).

*SLC7A11* is a protein-coding gene that encodes a sodium-independent amino acid transport system that mediates cysteine and glutamate transport. *SLC7A11* is a key component of the cystine/glutamate exchange system xc−, crucial in maintaining high glutathione levels within cells. Structurally, *SLC7A11* comprises 12 putative transmembrane domains, possesses a molecular weight of 55 kDa, and exhibits high hydrophobicity and substrate-specificity (Lim and Donaldson, 2011). Functionally, the system x_c_^−^ negatively regulates ferroptosis (Xie et al., 2016). Inhibition of *SLC7A11* reduces cystine uptake and compromises the antioxidant defenses of cells, which induces ferroptosis (Dixon et al., 2012; Lang et al., 2019). Unlike other cell death processes such as necrosis, autophagy, apoptosis, and others, ferroptosis is an oxidizing mechanism that requires iron (Tang et al., 2019). The buildup of toxic lipid reactive oxygen species during ferroptosis is most likely a result of the suppression of glutathione synthesis or the action of glutathione peroxidase 4, which deactivates cellular glutathione-dependent antioxidant defenses (Dixon et al., 2012; Cao and Dixon, 2016; Yang et al., 2014).

Considering the increased metabolic demand for iron and lipids in cancer cells, they exhibit a high susceptibility to ferroptosis. Furthermore, ferroptosis makes cancer cells more susceptible to radiation therapy and improves the efficacy of certain chemotherapeutic drugs (Xie et al., 2016; Li and Li, 2020). Cancer cells with high *SLC7A11* expression exhibit increased sensitivity under limited glucose conditions, leading to an increased dependence of cancer cells on glucose (Koppula et al., 2017). By investigating the correlation between SLC7A11 expression and immune cell infiltration in HNSCC, we aimed to uncover potential mechanisms through which SLC7A11 may influence the tumor microenvironment. Our findings suggest that SLC7A11 expression is significantly correlated with the infiltration levels of several immune cell types, such as T cells, B cells, and macrophages. These results provide valuable insights into the role of SLC7A11 in modulating immune responses within the tumor microenvironment, highlighting its potential as a therapeutic target for enhancing anti-tumor immunity in HNSCC (Dai et al., 2020). This research direction holds promise for developing more effective diagnostic and therapeutic approaches for HNSCC, ultimately improving patient prognosis and quality of life. We also noticed a positive correlation between *SLC7A11* expression and eosinophil infiltration, which was inversely correlated with CD8^+^ T-cell infiltration. CD8^+^ T cells are the main producers of interferon-γ, which suppresses the expression of SLC7A11, thereby promoting the ferroptosis and the peroxidation of lipids in tumor cells (Wang et al., 2019). Furthermore, in the current study, we observed that the high *SLC7A11* expression group was considerably enriched in signaling pathways, such as p53. This conclusion is in accordance with that of a previous study which showed that reducing *SLC7A11* expression decreases the expression of p53, which affects cystine absorption and makes cells more susceptible to ferroptosis (Imai et al., 2017). Furthermore, p53 participates in tumor suppression by mediating cell cycle arrest, apoptosis, aging, and metabolic regulation (Jiang et al., 2015; Bieging et al., 2014). Therefore, our study reflects the molecular mechanisms of the tumor microenvironment from an imaging perspective, providing new methods for evaluating tumor heterogeneity.

Most patients with HNSCC are diagnosed during the advanced disease stage without an obvious precancerous lesion. Given the high heterogeneity of HNSCC, prognostic information is crucial for clinical decision-making. *SLC7A11* interacts with tumor stem cell markers and has a negative impact on the prognosis in patients with HNSCC. Importantly, our Kaplan–Meier curve analysis showed that early high expression correlated with reduced patient survival, and the multivariate analysis identified high *SLC7A11* expression as a risk factor for poor OS (*p* = 0.047). Therefore, we developed a radiomics-based model to predict *SLC7A11* expression to facilitate clinical decision-making.

Radiomics is an application of artificial intelligence in the medical field that enables the prediction of disease prognosis. Radiomics possesses unique technical advantages for exploring the molecular mechanisms underlying diseases and evaluating treatment outcomes. Unlike naked-eye assessments or other quantitative imaging methods, radiomics can digitize tissue phenotypic features across different spatial scales (Chow, 2020). For example, Schniering et al. used CT radiomics to systematically analyze risk factors for interstitial lung disease development in humans and mice and successfully predicted progression-free survival rates (Schniering et al., 2022). Sun et al. (2018) successfully predicted biomarkers of CD8^+^ T-cell expression in tumor-infiltrating lymphocytes using an enhanced CT radiomics model for patients receiving PD-1 or PD-L1 monoclonal antibody immunotherapy. The authors demonstrated the effectiveness of machine learning in evaluating tumor prognoses (Sun et al., 2018). Radiomics involves the use of machine learning techniques including Bayesian models, random forests, decision trees, SVM, logical regression and deep learning more and more (Peng et al., 2021). In our study, both the SVM and LR models demonstrated excellent predictive performance for HNSCC. We identified optimal feature sets using a recursive feature elimination algorithm and established SVM and LR models to predict *SLC7A11* expression. These models consistently performed well in predicting gene overexpression probabilities and true values. The decision curve analysis displayed high clinical utility, and no discernible statistical variations were observed in the AUCs between the training and validation sets, indicating good model fitness. The DeLong test demonstrated that the SVM and LR models had strong prediction performance and revealed a substantial difference in their AUC values between the training and validation sets. When assessing the extensive efficacy of the radiomics model for prediction by applying the Brier score, a smaller value indicates better model prediction consistency. The SVM model had a higher Brier score than the LR model. Therefore, we selected the LR model to predict gene expression probabilities and RAD scores. The Wilcoxon test indicated statistically significant variations in RAD scores over high- and low-gene-expression groups. The high SLC7A11 expression group showed a low RAD score. Our radionics-based predictive model for SLC7A11 expression showed good efficacy and is valuable in guiding clinical prognosis.

Imaging changes are macroscopic manifestations of changes in microscopic components (molecules, cells, etc.). The changes in SLC7A11 molecules are highly likely to be the molecular pathological basis behind radiomics, and may show great potential in tumor treatment. Overexpression of SLC7A11 has been reported in various cancers, including lung, breast, and ovarian cancers, where it contributes to tumor growth, chemoresistance, and poor prognosis (Liu et al., 2020). In addition, the SLC7A11 inhibitor targeted therapy drug Agilvax is also in the development process. However, currently SLC7A11 detection is usually invasive, expensive, and based on local tumor tissue, which not only cannot represent the overall situation of the tumor, but also makes it difficult to achieve dynamic observation. Therefore, based on the overall tumor, this study constructed a non-invasive radiomics prediction model for SLC7A11, which can achieve prediction of patient prognosis. Non invasive prediction provides the possibility for dynamic monitoring of SLC7A11, lays the foundation for predicting SLC7A11 related treatments, and in the future, can also screen potential beneficiaries for SLC7A11 targeted therapy.

## 5 Conclusion

In patients with HNSCC, SLC7A11 expression substantially correlated with prognosis. Furthermore, our study demonstrated that an enhanced CT radiomics model, based on selected radiomics features, accurately predicted *SLC7A11* expression with high stability and diagnostic efficiency. As big data and precision medicine evolve, integrating radiomics with other omics is expected to emerge as a promising new Frontier for future research.

## Data Availability

The datasets presented in this study can be found in online repositories. The names of the repository/repositories and accession number(s) can be found in the article/Supplementary Material.

## Abbreviations

AUC, area under the receiver operating characteristic curve; CT, computed tomography; HNSCC, head and neck squamous cell carcinoma; KEGG, Kyoto Encyclopedia of Genes and Genomes; LR, logistic regression; MRI, magnetic resonance imaging; NRF2, nuclear factor-erythroid 2; OS, overall survival; PD-L1, programmed death ligand 1; m, messenger; ROC, receiver operating characteristic; SLC7A11, solute carrier family 7, member 11; SVM, support vector machines.

## References

[B1] Bieging K. T. Mello S. S. Attardi L. D. (2014). Unravelling mechanisms of p53-mediated tumour suppression. Nat. Rev. Cancer. 14, 359–370. 10.1038/nrc3711 24739573 PMC4049238

[B2] Bray F. Ferlay J. Soerjomataram I. Siegel R. L. Torre L. A. Jemal A. (2018). Global cancer statistics 2018: GLOBOCAN estimates of incidence and mortality worldwide for 36 cancers in 185 countries. CA Cancer J. Clin. 68, 394–424. 10.3322/caac.21492 30207593

[B3] Cao J. Y. Dixon S. J. (2016). Mechanisms of ferroptosis. Cell. Mol. Life Sci. 73, 2195–2209. 10.1007/s00018-016-2194-1 27048822 PMC4887533

[B4] Chow L. Q. M. (2020). Head and neck cancer. N. Engl. J. Med. 382, 60–72. 10.1056/NEJMra1715715 31893516

[B5] Cramer J. D. Burtness B. Le Q. T. Ferris R. L. (2019). The changing therapeutic landscape of head and neck cancer. Nat. Rev. Clin. Oncol. 16, 669–683. 10.1038/s41571-019-0227-z 31189965

[B6] Dai E. Zhu Z. Wahed S. Qu Z. Storkus W. J. Guo Z. S. (2020). Epigenetic modulation of antitumor immunity for improved cancer immunotherapy. Mol. Cancer 19 (1), 1–15. 10.1186/s12943-021-01464-x 34930302 PMC8691037

[B7] Dixon S. J. Lemberg K. M. Lamprecht M. R. Skouta R. Zaitsev E. M. Gleason C. E. (2012). Ferroptosis: an iron-dependent form of nonapoptotic cell death. Cell 149, 1060–1072. 10.1016/j.cell.2012.03.042 22632970 PMC3367386

[B8] Feng L. Zhao K. Sun L. Yin X. Zhang J. Liu C. (2021). SLC7A11 regulated by NRF2 modulates esophageal squamous cell carcinoma radiosensitivity by inhibiting ferroptosis. J. Transl. Med. 19, 367. 10.1186/s12967-021-03042-7 34446045 PMC8393811

[B9] He Q. Liu M. Huang W. Chen X. Zhang B. Zhang T. (2021). IL-1β-induced elevation of solute carrier family 7 member 11 promotes hepatocellular carcinoma metastasis through up-regulating programmed death ligand 1 and colony-stimulating factor 1. Hepatology 74, 3174–3193. 10.1002/hep.32062 34288020

[B10] Imai H. Matsuoka M. Kumagai T. Sakamoto T. Koumura T. (2017). Lipid peroxidation-dependent cell death regulated by GPx4 and ferroptosis. Curr. Top. Microbiol. Immunol. 403, 143–170. 10.1007/82_2016_508 28204974

[B11] Jiang L. Kon N. Li T. Wang S. J. Su T. Hibshoosh H. (2015). Ferroptosis as a p53-mediated activity during tumour suppression. Nature 520, 57–62. 10.1038/nature14344 25799988 PMC4455927

[B12] Johnson D. E. Burtness B. Leemans C. R. Lui V. W. Y. Bauman J. E. Grandis J. R. (2020). Head and neck squamous cell carcinoma. Nat. Rev. Dis. Prim. 6, 92. 10.1038/s41572-020-00224-3 33243986 PMC7944998

[B13] Kaleeba J. A. Berger E. A. (2006). Kaposi’s sarcoma-associated herpesvirus fusion-entry receptor: cystine transporter xCT. Science. 311, 1921–1924. 10.1126/science.1120878 16574866

[B14] Koppula P. Zhang Y. Shi J. Li W. Gan B. (2017). The glutamate/cystine antiporter SLC7A11/xCT enhances cancer cell dependency on glucose by exporting glutamate. J. Biol. Chem. 292, 14240–14249. 10.1074/jbc.M117.798405 28630042 PMC5572906

[B15] Koppula P. Zhuang L. Gan B. (2021). Cystine transporter SLC7A11/xCT in cancer: ferroptosis, nutrient dependency, and cancer therapy. Protein Cell 12, 599–620. 10.1007/s13238-020-00789-5 33000412 PMC8310547

[B16] Lang X. Green M. D. Wang W. Yu J. Choi J. E. Jiang L. (2019). Radiotherapy and immunotherapy promote tumoral lipid oxidation and ferroptosis via synergistic repression of SLC7A11. Cancer Discov. 9, 1673–1685. 10.1158/2159-8290.CD-19-0338 31554642 PMC6891128

[B17] Leemans C. R. Snijders P. J. F. Brakenhoff R. H. (2018). The molecular landscape of head and neck cancer. Nat. Rev. Cancer. 18, 269–282. 10.1038/nrc.2018.11 29497144

[B18] Li D. Li Y. (2020). The interaction between ferroptosis and lipid metabolism in cancer. Signal Transduct. Target. Ther. 5, 108. 10.1038/s41392-020-00216-5 32606298 PMC7327075

[B19] Lim J. C. Donaldson P. J. (2011). Focus on molecules: the cystine/glutamate exchanger (System x(c)(−)). Exp. Eye Res. 92, 162–163. 10.1016/j.exer.2010.05.007 20488177

[B20] Lin W. Wang C. Liu G. Bi C. Wang X. Zhou Q. (2020). SLC7A11/xCT in cancer: biological functions and therapeutic implications. Am. J. Cancer Res. 10, 3106–3126. 33163260 PMC7642655

[B21] Liu X. Olszewski K. Zhang Y. Lim E. W. Shi J. Zhang X. (2020). Cystine transporter regulation of pentose phosphate pathway dependency and disulfide stress exposes a targetable metabolic vulnerability in cancer. Nat. Cell Biol. 22 (4), 476–486. 10.1038/s41556-020-0496-x 32231310 PMC7194135

[B22] Peng Z. Wang Y. Wang Y. Jiang S. Fan R. Zhang H. (2021). Application of radiomics and machine learning in head and neck cancers. Int. J. Biol. Sci. 17, 475–486. 10.7150/ijbs.55716 33613106 PMC7893590

[B23] Schniering J. Maciukiewicz M. Gabrys H. S. Brunner M. Blüthgen C. Meier C. (2022). Computed tomography-based radiomics decodes prognostic and molecular differences in interstitial lung disease related to systemic sclerosis. Eur. Respir. J. 59, 2004503. 10.1183/13993003.04503-2020 34649979 PMC9117734

[B25] Sun R. Limkin E. J. Vakalopoulou M. Dercle L. Champiat S. Han S. R. (2018). A radiomics approach to assess tumour-infiltrating CD8 cells and response to anti-PD-1 or anti-PD-L1 immunotherapy: an imaging biomarker, retrospective multicohort study. Lancet Oncol. 19, 1180–1191. 10.1016/S1470-2045(18)30413-3 30120041

[B26] Tang D. Kang R. Berghe T. V. Vandenabeele P. Kroemer G. (2019). The molecular machinery of regulated cell death. Cell Res. 29, 347–364. 10.1038/s41422-019-0164-5 30948788 PMC6796845

[B27] Wang W. Green M. Choi J. E. Gijón M. Kennedy P. D. Johnson J. K. (2019). CD8+ T cells regulate tumour ferroptosis during cancer immunotherapy. Nature 569, 270–274. 10.1038/s41586-019-1170-y 31043744 PMC6533917

[B28] Xie Y. Hou W. Song X. Yu Y. Huang J. Sun X. (2016). Ferroptosis: process and function. Cell Death Differ. 23, 369–379. 10.1038/cdd.2015.158 26794443 PMC5072448

[B29] Yang W. S. Sriramaratnam R. Welsch M. E. Shimada K. Skouta R. Viswanathan V. S. (2014). Regulation of ferroptotic cancer cell death by GPX4. Cell 156, 317–331. 10.1016/j.cell.2013.12.010 24439385 PMC4076414

[B30] Zhou L. Q. Hu Y. Xiao H. J. (2021). The prognostic significance of survivin expression in patients with HNSCC: a systematic review and meta-analysis. BMC Cancer 21, 424. 10.1186/s12885-021-08170-3 33863308 PMC8052826

